# 6-Methyl­pyridine-2(1*H*)-thione

**DOI:** 10.1107/S1600536810014273

**Published:** 2010-04-24

**Authors:** Guo-Jun Lian, Bo Chen, Ting-Ting Zhang, Li-Zhen Zhuang, Hong-Ze Liang

**Affiliations:** a School of Public Health of Wenzhou Medical College, Wenzhou 325035, People’s Republic of China; b State Key Laboratory Base of Novel Functional Materials and Preparation Science, Faculty of Materials Science and Chemical Engineering, Ningbo University, Ningbo 315211, People’s Republic of China

## Abstract

There are two unique mol­ecules in the asymmetric unit of the title pyridine­thione derivative, C_6_H_7_NS, each of which adopts the thione rather than the mercaptan form. The rings in both mol­ecules are essentially planar, with maximum deviations from the least-squares planes through all non-H atoms of 0.021 (2) and 0.017 (2) Å. In the crystal structure, the mol­ecules form centrosymmetric cyclic dimers through inter­molecular N—H⋯S hydrogen bonds. Additional C—H(meth­yl)⋯S inter­actions generate a three-dimensional network.

## Related literature

For the synthesis of 2-mercaptopyridines, see: Thirtle (1946 ▶). For background to the applications of organic sulfur-containing compounds, see: Cui *et al.* (2009 ▶); Saadat *et al.* (2004 ▶); Qian *et al.* (2007 ▶). For metal complexes of 2-mercapto pyridine *N*-oxide and 6-methyl substituted derivatives, see: Hamaguchi *et al.* (2007 ▶); Chunchuryukin *et al.* (2006 ▶); Cotton *et al.* (1978 ▶); West *et al.* (1998 ▶); Fielding *et al.* (1997 ▶); Berardini *et al.* (1997 ▶); Tylicki *et al.* (1995 ▶); Hong *et al.* (1999 ▶); Cabeza *et al.* (2007 ▶).
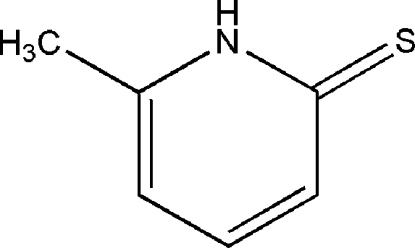


## Experimental

### 

#### Crystal data

C_6_H_7_NS
*M* *_r_* = 125.19
Monoclinic, 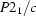

*a* = 7.4608 (15) Å
*b* = 14.902 (3) Å
*c* = 11.665 (2) Å
β = 94.85 (3)°
*V* = 1292.3 (4) Å^3^
*Z* = 8
Mo *K*α radiation
μ = 0.39 mm^−1^
*T* = 295 K
0.33 × 0.33 × 0.20 mm

#### Data collection

Rigaku R-AXIS RAPID diffractometer
Absorption correction: multi-scan (*ABSCOR*; Higashi, 1995 ▶) *T*_min_ = 0.880, *T*_max_ = 0.926
12472 measured reflections
2944 independent reflections
2088 reflections with *I* > 2σ(*I*)
*R*_int_ = 0.030

#### Refinement

*R*[*F* ^2^ > 2σ(*F* ^2^)] = 0.043
*wR*(*F* ^2^) = 0.148
*S* = 1.12
2944 reflections
146 parameters
H-atom parameters constrained
Δρ_max_ = 0.36 e Å^−3^
Δρ_min_ = −0.26 e Å^−3^

### 

Data collection: *RAPID-AUTO* (Rigaku, 1998 ▶); cell refinement: *RAPID-AUTO*; data reduction: *CrystalStructure* (Rigaku/MSC, 2004 ▶); program(s) used to solve structure: *SHELXS97* (Sheldrick, 2008 ▶); program(s) used to refine structure: *SHELXL97* (Sheldrick, 2008 ▶); molecular graphics: *SHELXTL* (Sheldrick, 2008 ▶); software used to prepare material for publication: *SHELXL97*.

## Supplementary Material

Crystal structure: contains datablocks global, I. DOI: 10.1107/S1600536810014273/sj2766sup1.cif

Structure factors: contains datablocks I. DOI: 10.1107/S1600536810014273/sj2766Isup2.hkl

Additional supplementary materials:  crystallographic information; 3D view; checkCIF report

## Figures and Tables

**Table 1:** Hydrogen-bond geometry (Å, °)

*D*—H⋯*A*	*D*—H	H⋯*A*	*D*⋯*A*	*D*—H⋯*A*
N1—H1*A*⋯S1^i^	0.86	2.50	3.3376 (19)	165
N2—H2*A*⋯S2^ii^	0.86	2.50	3.340 (2)	166
C1—H1*B*⋯S1^i^	0.96	2.79	3.678 (3)	154
C7—H7*A*⋯S2^ii^	0.96	2.74	3.639 (3)	156

Symmetry codes: (i) 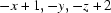; (ii) 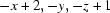.

**Fig. 1.**
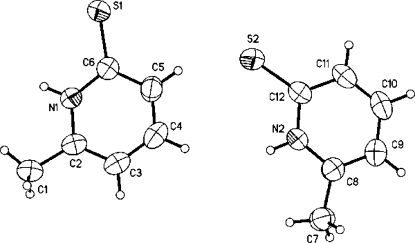
A perspective view of the title compound. Displacement ellipsoids are drawn at the 45% probability level.

**Fig. 2.**
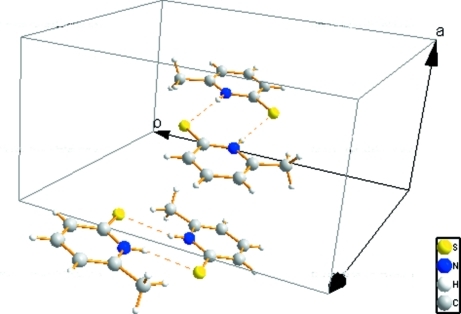
Part of the packing of the title compound showing the formation of centrosymmetric dimers.

## supplementary crystallographic information

### Comment

Organic sulfur-containing compounds have been frequently encountered or used in chemical industry (Cui *et al.*,  2009), life science (Saadat *et al.*,  2004) and pharmacy (Qian *et al.*,  2007).  Some of them have found their applications in crystal engineering. 2-mercaptopyridine, its 6-methyl substituted and N-oxide derivatives have exhibited rich coordination motifs. They can serve as monodentate (Hamaguchi *et al.*, 2007; Chunchuryukin *et al.*, 2006), bidentate (Cotton *et al.*, 1978; West *et al.*, 1998; Fielding *et al.*, 1997; Berardini *et al.*, 1997), and bridging (Tylicki *et al.*, 1995; Hong, *et al.*, 1999; Cabeza, *et al.*, 2007) ligands to coordinate metals. Though syntheses and crystal structures of these metal complexes have been reported, the crystal structure of 2-mercapto-6-methylpyridine itself has not been yet reported. Here we report its crystal structure and packing pattern.

A perspective view of the title compound is shown in Fig. 1. The C—S bond lengths were 1.694 (3) and 1.700 (2) Å, shorter than those in the above-mentioned metal complexes (typically 1.740 Å). This clearly indicates that the neutral title compound in solid state exists as a pyridinethione, while in metal complexes it ligates to metal centers as a pyridinethiolate anion. As shown in Fig. 2, adjacent two molecules are linked by intermolecular N–H···S interactions, forming a cyclic dimer.

### Experimental

6-methylpyridine-2(1H)-thione was synthesized by a literature method (Thirtle 1946).   X-ray quality single crystals were grown from ethyl acetate and petroleum ether (1/10, v/v).

### Refinement

H atoms bonded to C atoms were placed in geometrically calculated position and were refined using a riding model, the C–H bond lengths are 0.93 or 0.96 [Uiso(H) = 1.2 Ueq(C)] or [U_iso_(H) = 1.5 U_eq_(C)] for the methyl group, H atoms bonded to N atoms were placed in geometrically calculated positions and were also refined as riding [U_iso_(H) = 1.2 U_eq_(N)]. .

### Crystal data

**Table:**

C_6_H_7_NS	*F*(000) = 528
*M**_r_* = 125.19	*D*_x_ = 1.287 Mg m^−^^3^
Monoclinic, *P*2_1_/*c*	Mo *K*α radiation, λ = 0.71073 Å
Hall symbol: -P 2ybc	Cell parameters from 12782 reflections
*a* = 7.4608 (15) Å	θ = 3.1–27.4°
*b* = 14.902 (3) Å	µ = 0.39 mm^−^^1^
*c* = 11.665 (2) Å	*T* = 295 K
β = 94.85 (3)°	Block, yellow
*V* = 1292.3 (4) Å^3^	0.33 × 0.33 × 0.20 mm
*Z* = 8	

### Data collection

**Table:**

Rigaku R-AXIS RAPID diffractometer	2944 independent reflections
Radiation source: fine-focus sealed tube	2088 reflections with *I* > 2σ(*I*)
graphite	*R*_int_ = 0.030
Detector resolution: 0 pixels mm^-1^	θ_max_ = 27.4°, θ_min_ = 3.1°
ω scans	*h* = −9→9
Absorption correction: multi-scan (*ABSCOR*; Higashi, 1995)	*k* = −19→19
*T*_min_ = 0.880, *T*_max_ = 0.926	*l* = −15→15
12472 measured reflections	

### Refinement

**Table:**

Refinement on *F*^2^	Primary atom site location: structure-invariant direct methods
Least-squares matrix: full	Secondary atom site location: difference Fourier map
*R*[*F*^2^ > 2σ(*F*^2^)] = 0.043	Hydrogen site location: inferred from neighbouring sites
*wR*(*F*^2^) = 0.148	H-atom parameters constrained
*S* = 1.12	*w* = 1/[σ^2^(*F*_o_^2^) + (0.0738*P*)^2^ + 0.3029*P*] where *P* = (*F*_o_^2^ + 2*F*_c_^2^)/3
2944 reflections	(Δ/σ)_max_ < 0.001
146 parameters	Δρ_max_ = 0.36 e Å^−^^3^
0 restraints	Δρ_min_ = −0.26 e Å^−^^3^

### Special details

**Table:**

Geometry. All esds (except the esd in the dihedral angle between two l.s. planes) are estimated using the full covariance matrix. The cell esds are taken into account individually in the estimation of esds in distances, angles and torsion angles; correlations between esds in cell parameters are only used when they are defined by crystal symmetry. An approximate (isotropic) treatment of cell esds is used for estimating esds involving l.s. planes.
Refinement. Refinement of *F*^2^ against ALL reflections. The weighted *R*-factor wR and goodness of fit *S* are based on *F*^2^, conventional *R*-factors *R* are based on *F*, with *F* set to zero for negative *F*^2^. The threshold expression of *F*^2^ > σ(*F*^2^) is used only for calculating *R*-factors(gt) etc. and is not relevant to the choice of reflections for refinement. *R*-factors based on *F*^2^ are statistically about twice as large as those based on *F*, and *R*- factors based on ALL data will be even larger.

### Fractional atomic coordinates and isotropic or equivalent isotropic displacement parameters (Å^2^)

**Table:**

	*x*	*y*	*z*	*U*_iso_*/*U*_eq_	
S1	0.52754 (9)	0.10281 (4)	0.87258 (5)	0.0561 (2)	
S2	0.90012 (12)	0.13575 (4)	0.50014 (6)	0.0743 (3)	
N1	0.5873 (2)	−0.07318 (12)	0.85963 (15)	0.0470 (4)	
H1A	0.5725	−0.0723	0.9319	0.056*	
N2	0.9262 (2)	0.02468 (12)	0.32400 (15)	0.0488 (4)	
H2A	0.9555	−0.0139	0.3771	0.059*	
C1	0.6385 (5)	−0.23318 (17)	0.8905 (2)	0.0746 (8)	
H1B	0.6160	−0.2149	0.9669	0.112*	
H1C	0.7568	−0.2587	0.8916	0.112*	
H1D	0.5509	−0.2772	0.8633	0.112*	
C2	0.6257 (3)	−0.15375 (16)	0.8125 (2)	0.0541 (6)	
C3	0.6503 (4)	−0.15630 (18)	0.6979 (2)	0.0626 (6)	
H3A	0.6773	−0.2102	0.6631	0.075*	
C4	0.6350 (4)	−0.0782 (2)	0.6339 (2)	0.0658 (7)	
H4A	0.6513	−0.0800	0.5557	0.079*	
C5	0.5961 (3)	0.00181 (18)	0.68372 (19)	0.0593 (6)	
H5A	0.5868	0.0536	0.6392	0.071*	
C6	0.5702 (3)	0.00646 (15)	0.80167 (18)	0.0469 (5)	
C7	0.9694 (4)	−0.10058 (16)	0.1955 (2)	0.0609 (6)	
H7A	0.9963	−0.1291	0.2687	0.091*	
H7B	1.0723	−0.1042	0.1516	0.091*	
H7C	0.8693	−0.1303	0.1546	0.091*	
C8	0.9229 (3)	−0.00394 (16)	0.21353 (19)	0.0499 (5)	
C9	0.8776 (3)	0.05620 (18)	0.1275 (2)	0.0590 (6)	
H9A	0.8744	0.0386	0.0508	0.071*	
C10	0.8365 (4)	0.14393 (18)	0.1558 (2)	0.0634 (7)	
H10A	0.8072	0.1853	0.0975	0.076*	
C11	0.8384 (3)	0.17037 (16)	0.2678 (2)	0.0581 (6)	
H11A	0.8074	0.2290	0.2849	0.070*	
C12	0.8870 (3)	0.10976 (15)	0.3583 (2)	0.0517 (5)	

### Atomic displacement parameters (Å^2^)

**Table:**

	*U*^11^	*U*^22^	*U*^33^	*U*^12^	*U*^13^	*U*^23^
S1	0.0712 (4)	0.0439 (3)	0.0542 (4)	0.0043 (3)	0.0104 (3)	0.0009 (2)
S2	0.1163 (7)	0.0487 (4)	0.0574 (4)	0.0151 (4)	0.0044 (4)	−0.0032 (3)
N1	0.0524 (11)	0.0453 (9)	0.0438 (9)	0.0011 (8)	0.0076 (7)	−0.0034 (8)
N2	0.0570 (11)	0.0408 (9)	0.0485 (10)	0.0023 (8)	0.0037 (8)	0.0057 (8)
C1	0.108 (2)	0.0494 (14)	0.0673 (16)	0.0165 (14)	0.0117 (15)	−0.0044 (12)
C2	0.0553 (14)	0.0508 (13)	0.0567 (13)	0.0033 (10)	0.0078 (10)	−0.0093 (10)
C3	0.0703 (17)	0.0623 (15)	0.0561 (13)	0.0028 (12)	0.0102 (11)	−0.0160 (12)
C4	0.0716 (17)	0.0807 (18)	0.0463 (12)	−0.0021 (14)	0.0114 (11)	−0.0101 (12)
C5	0.0671 (16)	0.0642 (15)	0.0468 (12)	−0.0044 (12)	0.0059 (10)	0.0038 (11)
C6	0.0424 (12)	0.0508 (12)	0.0475 (11)	−0.0029 (9)	0.0044 (8)	−0.0009 (9)
C7	0.0648 (16)	0.0570 (14)	0.0614 (14)	0.0012 (12)	0.0087 (11)	−0.0063 (11)
C8	0.0442 (12)	0.0530 (12)	0.0531 (12)	−0.0045 (9)	0.0073 (9)	−0.0001 (10)
C9	0.0590 (15)	0.0682 (16)	0.0502 (12)	−0.0028 (12)	0.0057 (10)	0.0077 (11)
C10	0.0646 (16)	0.0623 (15)	0.0619 (15)	−0.0042 (12)	−0.0030 (11)	0.0229 (12)
C11	0.0595 (15)	0.0423 (11)	0.0716 (15)	−0.0034 (10)	−0.0006 (11)	0.0120 (11)
C12	0.0534 (13)	0.0418 (11)	0.0601 (13)	−0.0022 (10)	0.0053 (10)	0.0047 (10)

### Geometric parameters (Å, °)

**Table:**

S1—C6	1.700 (2)	C4—C5	1.368 (4)
S2—C12	1.694 (3)	C4—H4A	0.9300
N1—C2	1.361 (3)	C5—C6	1.407 (3)
N1—C6	1.367 (3)	C5—H5A	0.9300
N1—H1A	0.8600	C7—C8	1.500 (3)
N2—C8	1.356 (3)	C7—H7A	0.9600
N2—C12	1.369 (3)	C7—H7B	0.9600
N2—H2A	0.8600	C7—H7C	0.9600
C1—C2	1.491 (4)	C8—C9	1.367 (3)
C1—H1B	0.9600	C9—C10	1.389 (4)
C1—H1C	0.9600	C9—H9A	0.9300
C1—H1D	0.9600	C10—C11	1.363 (4)
C2—C3	1.366 (3)	C10—H10A	0.9300
C3—C4	1.383 (4)	C11—C12	1.413 (3)
C3—H3A	0.9300	C11—H11A	0.9300
			
C2—N1—C6	125.47 (18)	N1—C6—C5	115.2 (2)
C2—N1—H1A	117.3	N1—C6—S1	120.41 (15)
C6—N1—H1A	117.3	C5—C6—S1	124.34 (19)
C8—N2—C12	125.61 (19)	C8—C7—H7A	109.5
C8—N2—H2A	117.2	C8—C7—H7B	109.5
C12—N2—H2A	117.2	H7A—C7—H7B	109.5
C2—C1—H1B	109.5	C8—C7—H7C	109.5
C2—C1—H1C	109.5	H7A—C7—H7C	109.5
H1B—C1—H1C	109.5	H7B—C7—H7C	109.5
C2—C1—H1D	109.5	N2—C8—C9	118.4 (2)
H1B—C1—H1D	109.5	N2—C8—C7	116.7 (2)
H1C—C1—H1D	109.5	C9—C8—C7	124.9 (2)
N1—C2—C3	118.1 (2)	C8—C9—C10	119.2 (2)
N1—C2—C1	117.3 (2)	C8—C9—H9A	120.4
C3—C2—C1	124.6 (2)	C10—C9—H9A	120.4
C2—C3—C4	119.6 (2)	C11—C10—C9	121.0 (2)
C2—C3—H3A	120.2	C11—C10—H10A	119.5
C4—C3—H3A	120.2	C9—C10—H10A	119.5
C5—C4—C3	121.0 (2)	C10—C11—C12	120.8 (2)
C5—C4—H4A	119.5	C10—C11—H11A	119.6
C3—C4—H4A	119.5	C12—C11—H11A	119.6
C4—C5—C6	120.7 (2)	N2—C12—C11	114.9 (2)
C4—C5—H5A	119.7	N2—C12—S2	120.08 (17)
C6—C5—H5A	119.7	C11—C12—S2	125.00 (19)

### Hydrogen-bond geometry (Å, °)

**Table:**

*D*—H···*A*	*D*—H	H···*A*	*D*···*A*	*D*—H···*A*
N1—H1A···S1^i^	0.86	2.50	3.3376 (19)	165
N2—H2A···S2^ii^	0.86	2.50	3.340 (2)	166
C1—H1B···S1^i^	0.96	2.79	3.678 (3)	154
C7—H7A···S2^ii^	0.96	2.74	3.639 (3)	156

Symmetry codes: (i) −*x*+1, −*y*, −*z*+2; (ii) −*x*+2, −*y*, −*z*+1.
